# Additional Value From Free-Text Diagnoses in Electronic Health Records: Hybrid Dictionary and Machine Learning Classification Study

**DOI:** 10.2196/49007

**Published:** 2024-01-17

**Authors:** Tarun Mehra, Tobias Wekhof, Dagmar Iris Keller

**Affiliations:** 1 Department for Medical Oncology and Hematology University Hospital of Zurich Zurich Switzerland; 2 Center of Economic Research ETH Zurich Zurich Switzerland; 3 Faculty of Medicine, University of Zurich Zurich Switzerland; 4 Emergency Department, University Hospital of Zurich Zurich Switzerland

**Keywords:** electronic health records, free text, natural language processing, NLP, artificial intelligence, AI

## Abstract

**Background:**

Physicians are hesitant to forgo the opportunity of entering unstructured clinical notes for structured data entry in electronic health records. Does free text increase informational value in comparison with structured data?

**Objective:**

This study aims to compare information from unstructured text-based chief complaints harvested and processed by a natural language processing (NLP) algorithm with clinician-entered structured diagnoses in terms of their potential utility for automated improvement of patient workflows.

**Methods:**

Electronic health records of 293,298 patient visits at the emergency department of a Swiss university hospital from January 2014 to October 2021 were analyzed. Using emergency department overcrowding as a case in point, we compared supervised NLP-based keyword dictionaries of symptom clusters from unstructured clinical notes and clinician-entered chief complaints from a structured drop-down menu with the following 2 outcomes: hospitalization and high Emergency Severity Index (ESI) score.

**Results:**

Of 12 symptom clusters, the NLP cluster was substantial in predicting hospitalization in 11 (92%) clusters; 8 (67%) clusters remained significant even after controlling for the cluster of clinician-determined chief complaints in the model. All 12 NLP symptom clusters were significant in predicting a low ESI score, of which 9 (75%) remained significant when controlling for clinician-determined chief complaints. The correlation between NLP clusters and chief complaints was low (*r*=−0.04 to 0.6), indicating complementarity of information.

**Conclusions:**

The NLP-derived features and clinicians’ knowledge were complementary in explaining patient outcome heterogeneity. They can provide an efficient approach to patient flow management, for example, in an emergency medicine setting. We further demonstrated the feasibility of creating extensive and precise keyword dictionaries with NLP by medical experts without requiring programming knowledge. Using the dictionary, we could classify short and unstructured clinical texts into diagnostic categories defined by the clinician.

## Introduction

Organizational challenges, such as overcrowding in emergency departments (EDs), directly impact patient outcomes. The digitization of health records offers an opportunity to integrate artificial intelligence (AI) into patient management. However, health care workers often prefer to write unstructured text rather than entering structured data [1,2]. This raises the question of how future electronic health records (EHRs) should be designed: what additional value does free text provide?

We propose adding an additional dimension alongside the classic predictive task performed with text—inference to infer characteristics from text entries. Most studies using text analysis with patient records show promising results in predicting patient outcomes, such as in-hospital mortality, unplanned re-admission after 30 days, and prolonged length of hospital stay [3,4]. The benefits of unstructured text in EHRs for the improvement of prediction models have been demonstrated, as underscored by the extensive review by Seinen et al [5]. Indeed, 20% of the trials that were reported were conducted within a hospital ED environment. However, the analysis of the reported studies focused on demonstrating an improvement in predicting clinical outcomes, such as death or rehospitalization. We extend this approach by using the text not primarily to predict outcomes but to explain the correlation of patient subgroups with clinical outcomes. For instance, we show if certain symptoms documented in the ED triage are associated with a higher probability of an inpatient stay. Our results indicate that the information captured by clinical text-based notes is complementary to traditional structured data and can provide clinicians with valuable information about patients.

Overcrowding in the ED is an important case in point where AI supporting the optimization of patient workflows may substantially improve outcomes. It is a recognized challenge facing many EDs worldwide [6,7], adversely impacting patient outcomes [8]. These negative effects are evident during ED resource overload, such as during the COVID-19 pandemic [9]. More recently, senior public health officials in England have attributed up to 500 excess deaths per week during the recent winter months to delays caused by National Health Service capacity constraints [10,11]. Therefore, electronically enabled targeted patient selection could help speed up triage and reduce ED overcrowding. However, the optimal structure of EHRs remains controversial, particularly because clinicians tend to prefer the flexibility of entering unstructured text to structured data entry [12].

By comparing data extracted from 2 fields—1 derived from a structured drop-down menu indicating leading symptoms for ED admission and the other containing unstructured text—we can demonstrate that free text contains additional information beyond structured data and that these 2 types of data complement each other. With our semisupervised topic allocation method, we demonstrate the ability to capture more comprehensive information about a patient’s symptom cluster compared with relying solely on a manually attributed single chief complaint. Moreover, we present a transparent approach for extracting topics from short clinical texts based on natural language processing (NLP)–supported annotated clinical libraries, which can be fed into predictive models. In addition to being transparent, our method is language independent and easy to implement for clinical researchers (although the dictionaries we constructed are in German, researchers can easily use our method to construct their own topic dictionaries in any language).

Our approach is based on constructing a dictionary with keywords that define a topic. In contrast to dictionary approaches, unsupervised topic models, such as the latent Dirichlet allocation [13], are often used. However, finding topics in short-text samples using these models is challenging [14]. Moreover, unsupervised models might not capture topics that are of interest to the researcher because these models differentiate between topics based on their statistical difference. For instance, it could be that latent Dirichlet allocation defines topics based on words about the age and gender of the patients because these are the most distinctive features. However, the researcher may be interested in the diagnosis, which is more challenging to classify.

In contrast, supervised machine learning methods require creating a manually classified training data set. The algorithm learns how to classify future data into topics based on the training set. When dealing with a high volume of topics, both human classification and the algorithm’s training run the risk of creating noise. Similarly, regression approaches for supervised classifications are not suitable for many topics. Therefore, we chose a dictionary approach based on keywords. To facilitate the selection of the keywords, we developed a preselection of words based on a measure of their semantic similarity. As our presorting of words uses word embeddings, we consider our approach as a hybrid between dictionary- and machine learning–based approaches [15].

Our approach, combined with clinical notes, allow us to address 2 questions:

- What additional information does the free text provide on the patient being admitted compared with the suspected diagnosis from the drop-down menu?
- Could this additional information be useful for clinical or organizational purposes?

## Methods

### Data

**Figure 1:**
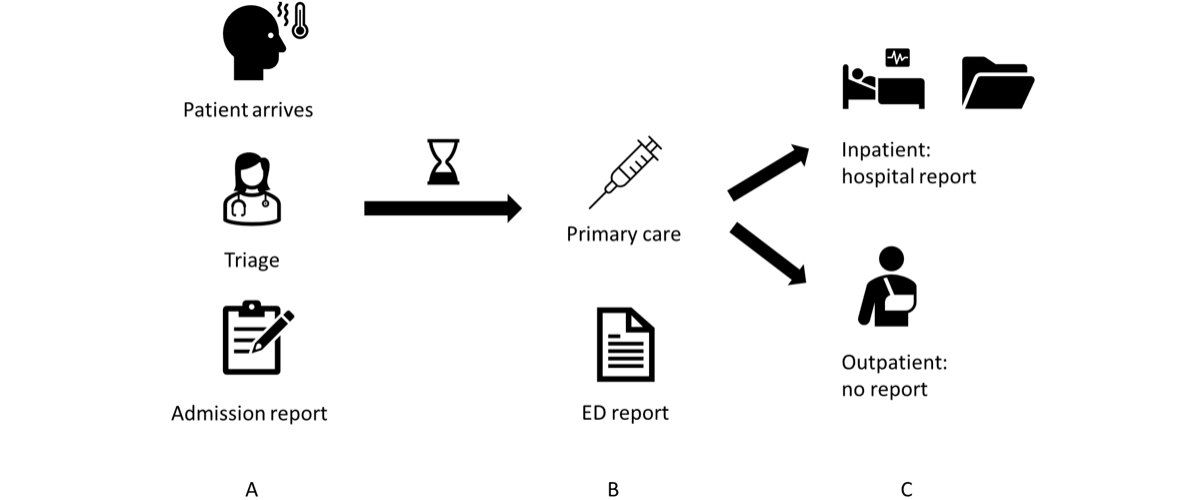
Patient flow in emergency department (ED) and associated reports.

We used data from the ED’s admission report. Figure 1 provides a contextual representation of this data type in relation to patient flow and other documents associated with patients. In step 1, patients present themselves at the ED and are admitted in the system. A medical professional conducts the triage by quickly assessing the main symptoms and their severity using the Emergency Severity Index (ESI) score, resulting in an admission report. This report is for the internal patient management within the ED and contains basic patient information (age, gender, and so on) along with the chief complaints and symptoms.

After a waiting time (which depends on the triage score), the patient receives primary care from a medical professional, which is documented in the ED report. The ED report summarizes the patient’s entire stay at the ED and is issued at the end of the patient care from the ED. In the third step, the patient is either discharged into ambulatory care (which does not create any further documents) or is transferred to inpatient care, which results in the classic medical records.

For our analysis, we used the first type of document: the internal ED admission report. Unlike the other types of documents, this report is issued before treatment and provides an opportunity to manage patient flow. Although the ED report from step 2 could also be used for inpatient management, this proves challenging in practice because inpatient care is very heterogeneous and depends on many factors, including different organizational structures in every hospital department. In contrast, the ED admission reports can be used for homogeneous organization within the ED.

Our initial data set contained 293,298 patient visits to the ED of the University Hospital of Zurich, Switzerland, from January 1, 2014, to October 31, 2021 (in German; received in the Excel [Microsoft Corporation] format). For each visit, the data set includes a short text from the triage with the patient’s symptoms, along with our 2 outcomes of interest (triage score “ESI,” which we further explain below, and type of discharge), basic patient characteristics (patient visit pseudo ID, age, gender, admission type [self, ambulance, or police], and admission reason [accident or illness]), ED organizational variables (average number of patients in ED; average patient waiting time; night, late, or early shift; and treating ED team [internal medicine, surgery, neurology, neurosurgery, or psychiatry]), and the visit’s time stamp. The summary statistics of these variables are presented in Table 1.

**Figure 2:**
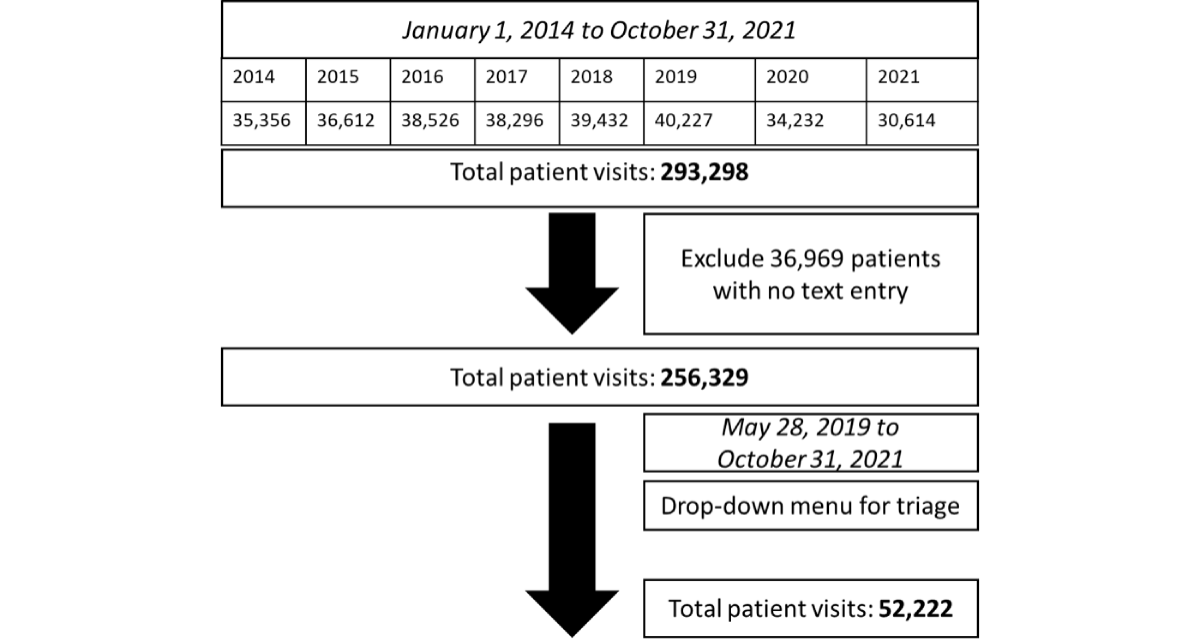
Data structure.

After excluding cases with no records in the string variable “suspected diagnosis” on admission on which NLP analysis was to be performed, the data set comprised 256,329 (87.4%) of the initial data set of 293,298 patient visits. We only used 2019 to 2021 for comparison as these visits had a recorded chief complaint, reducing the data set to the final sample of 52,222 patient visits. Patients directly admitted to the shock room (ie, ESI score=1) were not considered in our analysis, as no additional triage was performed upon admission. The data structure of our analysis is summarized in Figure 2, and the recorded variables are presented in Textbox 1.

The ESI is an internationally established 5-level triage algorithm widely used in EDs and is based on the acuity as well as the resource intensity of anticipated emergency care, with level 1 denoting acute life-threatening conditions, such as massive trauma warranting immediate, life-saving care, and level 5 denoting non–time-critical conditions of low complexity [13]. Cases triaged as ESI 4 or 5 (approximately 16% of patients) are usually fast-tracked to specialized treatment rooms because the medical resources required to treat these patients are low, and thus, they can be managed in parallel by a dedicated team, which reduces ED congestion. ESI 2 or 3 typically require a more thorough workup. Hence, for the outcome variable “low ESI,” we decided to set the cutoff at ESI<4, that is, patients with “low ESI” had been triaged with a score of 2 or 3. Furthermore, the data set included free-text fields (strings), namely, the suspected diagnosis at admission and the diagnosis at discharge.

In the admission process, the clinician performing triage records the patient’s symptoms in written form in 2 to 3 sentences. The purpose of this free text is to preregister the patient in the ED and enable all team members to become aware of the impending clinical problems. To our knowledge, all the larger EDs in German-speaking countries with full EHR note the reason for admission in the form of a short, unstructured text upon notification of a pending ED admission.

From May 28, 2019, onward, the symptoms were additionally recorded as so-called chief complaints from a drop-down menu (ordinal variable). The difference between the free text and the chief complaint was that the chief complaint was a fixed category selected from a drop-down menu and was primarily intended to serve administrative and statistical purposes, that is, to allow for post hoc analysis of the patient composition of the ED.

During the entire study period, the list of chief complaints (n=99) varied over time or contained doublets, which we grouped into 58 symptom topics. For patient visits with a selected chief complaint from the drop-down option “Diverse,” it was unclear if a leading symptom had been attributed at triage; hence, we did not include them in the list of chief complaints (referred to as lead symptoms [LS]). Furthermore, we grouped 5 chief complaints with very low occurrences, such as “drowning accident” or “flu vaccine,” into our class “diverse.” However, we did not use this group in further analysis because of the heterogeneity of the symptoms included. The lead symptom topics were then aggregated into 12 clusters by the authors according to clinical judgment. The complete list of LS can be found in Table S1 in Multimedia Appendix 1.

A total of 65 variables from 2014 to 2018 and 69 variables from 2019 to 2021 (including the chief complaint) were recorded in the initial data set. A total of 65 variables from 2014 to 2018 were constant throughout 2014 to 2021 and were retained for preprocessing. The final data table used for the analysis contained the variables listed in Table 1, in addition to the patient ID, year and weekday of the consultation derived from the admission time stamp, the treating ED team (internal medicine, surgery, neurology, or psychiatry), as well as the LS clusters from the drop-down menu and the NLP-extracted topic clusters that were obtained from the field “suspected diagnoses,” discussed in detail in *Analysis: Topic Allocation* section. In addition, the table contained the outcomes “inpatient” and “ESI score<4” as binary variables. Two further outcomes were considered, namely, readmission within 30 days and waiting time>30 minutes, but were discarded owing to doubts regarding the quality and consistency of the entered data. We retained the outcomes “inpatient” and “ESI score<4” owing to their direct association with the immediacy of the outcome in the patient pathway within the ED, ensuring robust data quality.

**Table 1:** Summary statistics of the patient population (n=52,222)^a^.

Variable	Values
Age (y), mean (SD)	46.5 (19.7)
Female, n (%)	23,782 (45.54)
Emergency Severity Index score (out of 5), mean (SD)	3.3 (0.6)
Fast track, n (%)	8264 (15.82)
Number of patients in the emergency department, mean (SD)	19.8 (8.3)
Early shift, n (%)	21,644 (41.45)
With emergency medical service, n (%)	9020 (17.27)
With police, n (%)	188 (0.36)
Accident, n (%)	16,845 (32.26)
Inpatient, n (%)	14,112 (27.02)
Night shift, n (%)	7915 (15.16)
Late shift, n (%)	22,663 (43.4)

^a^The total sample contains patient visits for the period from May 28, 2019, to October 31, 2021.

**Variables recorded for our analysis.**

**Triage**

- Suspected diagnosis (free text) and Emergency Severity Index score

**Type of discharge**

- Hospitalization, ambulatory treatment, or patient has run away

**Patient characteristics**

- Patient visit pseudo ID, age, gender, admission type (self, ambulance, or police), and admission reason (accident or illness)

**Organizational**

- Average number of patients in emergency department (ED); average patient waiting time; night, late, or early shift; and treating ED team (internal medicine, surgery, neurology, or psychiatry)

**Time**

- Time stamp

### Analysis: Topic Allocation

We selected the field “suspected diagnosis” to extract the symptoms or complaints that led to ED admission according to the oral report received by the ED physician in charge, as mentioned previously. This field comprises a short-text string entered by the ED physician upon receiving information about the patient’s expected arrival at the ED. This information can be transmitted to the ED physician by a referring physician or ambulance well in advance of a patient’s arrival. The text is entered before the patient triage is performed by the triage ED nurse. As a clinical note, the physician’s text entry is part of the EHR. The information contained in the string “suspected diagnoses” is supposed to be similar to the selected chief complaint from the drop-down menu “lead symptom.” Indeed, the latter variable was added later (in 2019) to facilitate the administrative analysis of causes for ED admission, as an analysis using unstructured text was not possible by the hospital administration. Both fields are supposed to contain the medical reason, or chief complaint, leading to ED admission.

We constructed a measure of the semantic distance of all words in the corpus by training a word embedding. Word embeddings are matrices in which each column represents a word and its relative distance to other words (eg, the distance between blood and red is smaller than that between blood and green). Hence, it is possible to find the most similar words for a given keyword using the smallest distance measured with the cosine similarity. To train the word embedding, we used word2vec with the entire text corpus and the continuous bag-of-words algorithm from the Python library Gensim [16], with an embedding size of 300 computed with 100 epochs.

**Figure 3:**
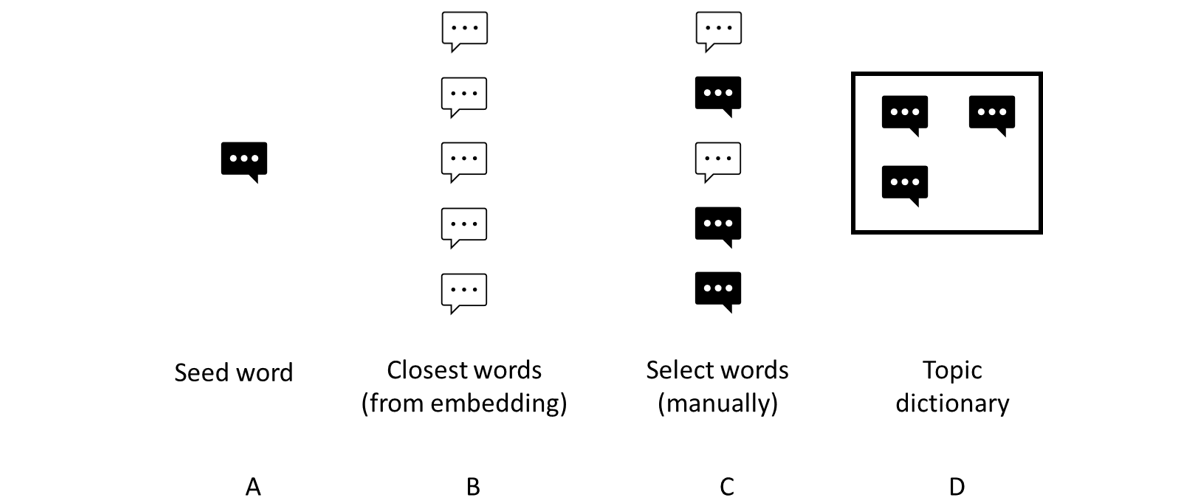
Topic dictionaries with semimanual keyword selection. (A) The researcher selects an initial seed word for a topic. (B) Using word embeddings, a list of semantically similar words from the corpus is generated. (C) The researcher manually selects words that are associated with the topic. (D) The topic dictionary is created.

To construct our topic dictionaries, we proceeded in 4 steps, as shown in Figure 3. First, we manually defined topics and selected between 2 and 20 initial seed words (henceforth “keywords”) by reading some of the texts and using prior medical knowledge. A smaller number of keywords were used for the design of the topic “infection” (n=1). A larger number of initial keywords were used for the design of the topics “intoxication” (n=40) and “skin” (n=28). In step 2, we then searched for up to 50 of the semantically closest words for each initial list. With the help of the word embedding, it is possible to search for the words that maximize the cosine similarity for the seed keywords. In addition, we only considered keywords that occurred at least 10 times. This list of similar words allowed us to efficiently increase the dictionary for each topic. In step 3, we manually chose words from the preselection of similar words to the seed word, resulting in a separate dictionary per topic (step 4). In some instances, the dictionary used combinations of words. For instance, the topic “chest pain” was allocated to combinations of words such as “pain” or “pressure” with the words “chest” or “thorax.”

This table presents the distribution of the diagnosis topics obtained with the NLP-based text annotation before and after the spherical feature annotation. The total number of cases was 52,222, and 20.38% could not be attributed with a diagnosis topic.

The summary of the increase in tags per topic cluster through the NLP-based expansion of our topics library is presented in Table 2. The first column shows the percentage of the sample tagged with a topic using the original keyword approach. The proportion of clinical topics ranged from 0.72% for COVID-19 to 31.6% for trauma-related visits. It should be noted that patient visits can be allocated with multiple topics. The next column shows the share of visits with the spherically increased dictionary, with the percentage increase in topic shares in the last column. Overall, the spherical dictionary enhancement decreased the number of nontagged visits by nearly 25%, from 27.08% of the sample to 20.24%. For the individual topics, the additional keywords increased their share, ranging from 5.29% for trauma to 286.35% for general administrative visits.

**Figure 4:**
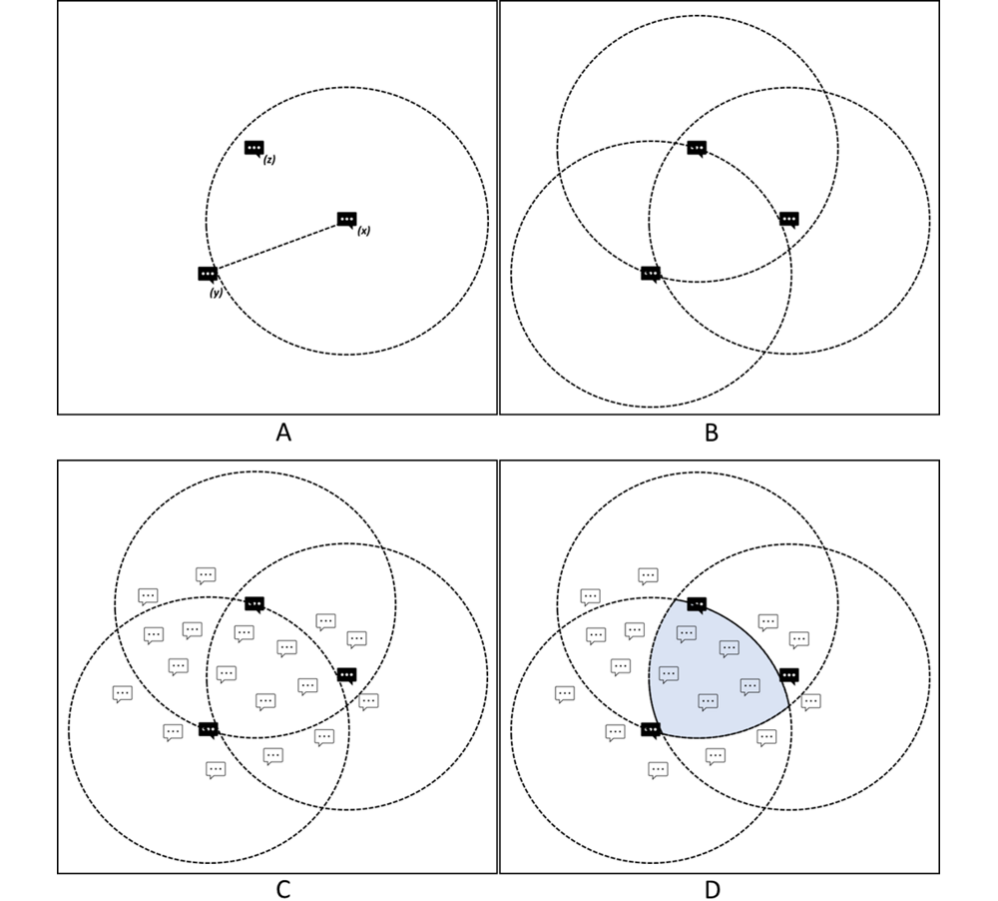
Spherical dictionary enhancement. (A) Step A uses the largest distance between 2 words that are already in the topic. The circle around the word (x) shows the region in the embedding space with words closer to (x) than the maximum distance. (B) The same region is circled around the other 2 words (y) and (z). (C) The other words in the embedding space that were initially not included in the topic. (D) The intersection of the 3 circles defines the area in the embedding space where the distance of each word is smaller than the maximum distance.

In the second procedure, we automatically increased the number of keywords for each topic dictionary. This process is shown in Figure 4, which can be imagined as constructing a multidimensional sphere using the initial keywords. The additional keywords were then located within that sphere.

The “spherical” dictionary enhancement consists of the following steps:

- Compute all distances between the keywords and retain the largest distance (ie, the distance between the 2 least similar words). For each keyword, this distance is the radius of a circle in the embedding space (steps 1 and 2).
- For each of the initial keywords, identify the n-closest words (not in the topic dictionary) using the cosine similarity (step 3).
- Retain these additional words if their distance to all other initial keywords is smaller than the maximum distance computed in the first step, that is, if the new words are in the intersection of all circles (step 4).

Using the abovementioned approach, we could tag 79.76% (41,653/52,222) of the final sample. The remaining texts could not be tagged because they either belonged to small topics that we did not define or because these texts did not contain words that are present in the dictionary.

Once the dictionaries for each topic are constructed, they can be used for additional patient visits and for similar data sets, which makes the approach easily scalable.

**Table 2:** Spherical feature annotation and increase in topic share (n=52,222)^a^.

Clinical topic NLP^b^	Records tagged initially, n (%)	Records tagged NLP-augmented, n (%)	Increase in tagged patient records, n (%)^c^
COVID-19	375 (0.72)	405 (0.78)	30 (8)
General symptom	6401 (12.26)	6867 (13.15)	466 (7.28)
General administration	315 (0.6)	1217 (2.33)	902 (286.35)
Systemic clinical	3219 (6.16)	3519 (6.74)	300 (9.32)
Gastrointestinal	3421 (6.55)	4159 (7.96)	738 (21.57)
Respiratory	4040 (6.55)	4159 (7.96)	738 (21.57)
Cardiovascular	2683 (5.14)	5219 (9.99)	2536 (94.52)
Neurological	414 (7.93)	4485 (8.59)	345 (8.33)
Eye; ear, nose, and throat; and derma	1818 (3.48)	2061 (3.95)	243 (13.37)
Gynecology and urology	2712 (5.19)	3004 (5.75)	292 (10.77)
Trauma	16,516 (31.63)	17,389 (33.3)	873 (5.29)
General psychiatric	1989 (3.81)	2627 (5.03)	638 (32.08)
No tag	14,141 (27.08)	10,569 (20.24)	–3572 (–25.26)

^a^This table presents the distribution of the diagnosis topics obtained with the NLP-based text annotation before and after the spherical feature annotation.

^b^NLP: natural language processing.

^c^Percent of initially recorded tags.

### Ethical Considerations

A waiver from the cantonal ethics committee was obtained before the commencement of this study (BASEC-Nr. Req-2019-00671).

## Results

In the first step, we performed a descriptive analysis of the topics. To this end, we first excluded cases without a manually selected LS for further analysis and obtained a data set with 52,222 entries. Of the 52,222 patient visits included in our final analysis, 5994 (11.48%) had a manually recorded chief complaint that was not otherwise specified (eg, “Diverse”) and could not be classified as a symptom Of the 52,222 entries, 10,569 (20.24%) were not tagged with an NLP topic.

The distribution of all NLP topics is shown in Table 3. The distribution ranged from 0.05% of patient visits tagged with the NLP topic “dementia” to 9.89% for “wound.” The largest cluster of aggregated NLP symptom-related topics was “trauma,” with 33.1% of visits, and the smallest was “COVID,” with 0.8% of visits. The distribution of chief complaints can be found in Table S1 in Multimedia Appendix 1. In total, the distribution ranged from 0.01% of patient visits for the recorded chief complaints “melaena,” “hearing problems,” and “contact with chemicals” to 14.6% for “COVID.” The largest cluster of aggregated chief complaints was “trauma” with 23.6% and the smallest was “general organizational” with 1.2% of visits.

For comparability, we grouped all LS and NLP topics into 12 identical symptom clusters, which can be found in Table 4.

**Table 3:** Clusters for natural language processing–extracted topics (n=52,222)^a^.

Cluster and subcluster detail			Values, n (%)
COVID-19			401 (0.77)
**General symptoms**			6852 (13.12)
	Fever	2440 (4.67)	
	Pain	4505 (8.63)	
	General weakness	80 (0.15)	
	Back pain	438 (0.84)	
**General organizational**			1217 (2.33)
	Follow-up and prescription	1217 (2.33)	
**Systemic**			3519 (6.74)
	Infection not otherwise specified	1239 (2.37)	
	Sepsis	125 (0.24)	
	Anaphylaxia and allergy	261 (0.5)	
	Cancer	1688 (3.23)	
	Transplantation	227 (0.43)	
	Glycemia	138 (0.26)	
**Gastrointestinal**			4147 (7.94)
	Gastrointestinal bleeding	522 (1)	
	Abdominal pain	1879 (3.6)	
	Diarrhea, vomiting, and nausea	2248 (4.3)	
**Respiratory**			4311 (8.26)
	Upper airway	1592 (3.05)	
	Lower airway	1934 (3.7)	
	Influenza	440 (0.84)	
	Dyspnea	2197 (4.21)	
**Cardiovascular**			5211 (9.98)
	Chest pain	3569 (6.83)	
	Palpitations and arrythmia	518 (0.99)	
	Pulmonary embolism	281 (0.54)	
	Deep venous thrombosis	528 (1.01)	
	Hypertension	394 (0.75)	
**Neurological**			4466 (8.55)
	Headache	1189 (2.28)	
	Neurological	1737 (3.33)	
	Vigilance and disorientation	191 (0.37)	
	Dementia	24 (0.05)	
	Syncope	453 (0.87)	
	Vertigo and dizziness	934 (1.79)	
	Convulsion	226 (0.43)	
**Eye; ear, nose, and throat; and skin**			2061 (3.95)
	Epistaxis	58 (0.11)	
	Eye symptoms	703 (1.35)	
	Hearing and auricular	18 (0.03)	
	Skin	1311 (2.51)	
**Urological and gynecological**			3004 (5.75)
	Urological and kidney	2973 (5.69)	
	Pregnancy	34 (0.07)	
**Trauma**			17,302 (33.13)
	Wound	5163 (9.89)	
	Fracture and luxation	5375 (10.29)	
	Trauma and head	2171 (4.16)	
	Burns	141 (0.27)	
	Fall	729 (1.4)	
	Trauma not otherwise specified	9278 (17.77)	
	Bleeding not otherwise specified	986 (1.89)	
	Collision	1250 (2.39)	
	Traffic	314 (0.6)	
**Psychiatric**			2625 (5.03)
	Intoxication	1146 (2.19)	
	Psychiatric	851 (1.63)	
	Fear	725 (1.39)	
**Severity**			
	Nonsevere	113 (0.22)	
	Severe	235 (0.45)	
	Chronic	55 (0.11)	
	Acute	232 (0.44)	

^a^This table presents the distribution of the diagnosis topics obtained with the natural language processing–based text annotation. In total, 20.38% of cases could not be attributed with a diagnosis topic.

**Table 4:** Summary statistics feature annotations (n=52,222)^a^.

Cluster	LS^b^, (n)	NLP^c^ (n)	Correlation (*r*)^d^	Consistency^e^
COVID-19	7623	401	0.18	0.05
General symptom	7993	6852	−0.04	0.10
General administration	642	1217	0.01	0.04
Systemic clinical	1983	3519	0.12	0.22
Gastrointestinal	4063	4147	0.41	0.46
Respiratory	872	4311	0.17	0.44
Cardiovascular	2245	5211	0.28	0.49
Neurological	5123	4466	0.44	0.46
Eye; ear, nose, and throat; and derma	1041	2061	0.26	0.39
Gynecology and urology	1206	3004	0.40	0.67
Trauma	12,337	17,302	0.54	0.79
General psychiatric	1610	2625	0.60	0.78
No tag	5994	10,644	0.07	0.28

^a^This table presents the number of tagged cases for each chief cluster with both the natural language processing–based method and based on the chief complaint tag.

^b^LS: lead symptom.

^c^NLP: natural language processing.

^d^Correlation between LS and NLP.

^e^The number of overlapping LS and NLP tags divided by the total number of LS tags.

In addition to the NLP symptom-related topics, 4 modulating NLP topics, “acute,” “chronic,” “nonsevere,” and “severe,” were recorded, also based on keywords (ie, words in the text indicating severity). The purpose of the modulating topics is to provide more information on severity and control for this dimension in the further analysis.

We found that the correlation between LS clusters and NLP clusters was low (Table 4). Similarly, consistency varies relative to the LS. We also calculated the consistency of the NLP tags relative to the LS groups (the LS groups are the denominator; being more established, we use them as a benchmark). For most clusters, the consistency is approximately 50%, with trauma and psychiatric diagnosis having the highest consistency of 78% and 79%, respectively, and general administration and COVID-19 having the lowest consistency of 4% and 5%, respectively.

Compared with the LS clusters, our NLP topics have the advantage that a patient visit can be tagged to multiple topics. Table S2 in Multimedia Appendix 1 shows the number of NLP topics for each LS cluster. Of the 46,228 patient visits where we could assign a manually recorded chief complaint, 8950 (19.36%) were not tagged with an NLP topic. In contrast, 33.48% (15,477/46,228) of the visits were tagged with at least 2 NLP topics.

We estimated 3 models using logistic regression to show the association of the different symptom groups with the ESI and inpatient indicators:

 Model 1: Y_i_ = α + βX_i_ + γZ_i_ + ε_i_  **(1)**

Model 2: Y_i_ = α + βX_i_ + δW_i_ + ε_i_  **(2)**

Model 3: Y_i_ = α + βX_i_ + γZ_i_ + δW_i_ + ε_i_  **(3)**

where *Y_i_* is either the ESI or inpatient indicator variable for patient visit *i*, *α* the intercept, *X_i_* is a vector of demographic and organizational variables for patient visit *I* (age; gender; admission type; admission reason; average number of patients in ED; average patient waiting time; night, late, or early shift; and treating ED team), *Z_i_* is a vector of the NLP-derived symptom clusters, *W*_i_ is a vector of the lead symptom–derived cluster (based on the drop-down menu), and *ε_i_* is the error term.

Tables 5 and 6 present the results. Column 1 shows the NLP-derived groups, with coefficients ranging between 5% and 13% increased or decreased probability of a high ESI score or 5% to 19% increased or decreased probability for hospitalization. The drop-down–based LS in column 2 has similar but slightly larger coefficients. Column 3 shows both variables, as in model 3, in this specification, the coefficients are mostly complementary, meaning that if a patient shows the same symptom in both the NLP and LS measures, the probabilities can be added. Note that this is not owing to multicollinearity because both coefficients remain significant in most cases.

**Table 5:** Linear probability model on “Inpatient”^a^.

Name of cluster^b^	Model 1^c^, regression coefficient (SE)	Model 2^c^, regression coefficient (SE)	Model 3 including both measures^d^, regression coefficient (SE)
NLP^e^ cluster: COVID-19	0.048^f^ (0.019)	N/A^g^	−0.022 (0.022)
Chief complaint cluster: COVID-19	N/A	0.127^g^ (0.007)	0.133^h^ (0.008)
NLP cluster: general symptoms	0.011^f^ (0.005)	N/A	−0.019^h^ (0.005)
Chief complaint cluster: general symptoms	N/A	−0.002 (0.007)	0.000 (0.007)
NLP cluster: general organizational	−0.004 (0.011)	N/A	0.006 (0.011)
Chief complaint cluster: general organizational	N/A	−0.062^g^ (0.016)	−0.052^h^ (0.016)
NLP cluster: systemic	0.117^h^ (0.007)	N/A	0.101^h^ (0.007)
Chief complaint cluster: systemic	N/A	0.118^h^ (0.010)	0.104^h^ (0.010)
NLP cluster: gastrointestinal	0.071^h^ (0.006)	N/A	0.040^h^ (0.007)
Chief complaint cluster: gastrointestinal	N/A	0.083^h^ (0.008)	0.059^h^ (0.008)
NLP cluster: respiratory	0.063^h^ (0.007)	N/A	−0.017^f^ (0.008)
Chief complaint cluster: respiratory	N/A	0.133^h^ (0.014)	0.126^f^ (0.014)
NLP cluster: cardiovascular	−0.020^h^ (0.006)	N/A	−0.009 (0.006)
Chief complaint cluster: cardiovascular	N/A	−0.038^h^ (0.010)	−0.031^h^ (0.010)
NLP cluster: neurological	−0.046^h^ (0.007)	N/A	−0.045^h^ (0.007)
Chief complaint cluster: neurological	N/A	−0.058^h^ (0.009)	−0.048^h^ (0.009)
NLP cluster: eye, ENT^i^, or skin	−0.055^h^ (0.009)	N/A	−0.044^h^ (0.009)
Chief complaint cluster: eye, ENT, or skin	N/A	−0.128^h^ (0.013)	−0.112^h^ (0.013)
NLP cluster: urological or gynecological	−0.015^f^ (0.008)	N/A	−0.004 (0.008)
Chief complaint cluster: urological or gynecological	N/A	−0.033^h^ (0.012)	−0.036^h^ (0.013)
NLP cluster: trauma	−0.041^h^ (0.005)	N/A	−0.038^h^ (0.005)
Chief complaint cluster: trauma	N/A	0.011 (0.007)	0.020^h^ (0.007)
NLP cluster: psychiatric	−0.079^h^ (0.009)	N/A	−0.053^h^ (0.010)
Chief complaint cluster: psychiatric	N/A	−0.068^h^ (0.013)	−0.039^h^ (0.014)

^a^This table presents the results from a linear probability model with inpatients as the dependent variable. All the models include a set of demographic and administrative covariates.

^b^Observation: 52,222; *R*^2^=0.259.

^c^Observation: 52,222; *R*^2^=0.263.

^d^Observation: 52,222; *R*^2^=0.269.

^e^NLP: natural language processing.

^f^*P*<.05.

^g^N/A: not applicable.

^h^*P*<.01.

^i^ENT: ear, nose, and throat.

**Table 6:** Linear probability model on “low Emergency Severity Index (ESI) score”^a^.

Name of cluster^b^	Model 1^c^, regression coefficient (SE)	Model 2^c^, regression coefficient (SE)	Model 3 including both measures^d^, regression coefficient (SE)
NLP^e^ cluster: COVID-19	0.079^f^ (0.019)	N/A^g^	0.023 (0.019)
Chief complaint cluster: COVID-19	N/A	0.214^f^ (0.007)	0.172^f^ (0.007)
NLP cluster: general symptoms	0.036^f^ (0.005)	N/A	−0.023^f^ (0.005)
Chief complaint cluster: general symptoms	N/A	−0.142^f^ (0.007)	0.127^f^ (0.007)
NLP cluster: general organizational	−0.050 (0.011)	N/A	−0.044^f^ (0.011)
Chief complaint cluster: general organizational	N/A	0.308^f^ (0.016)	0.352^f^ (0.016)
NLP cluster: systemic	0.076^f^ (0.007)	N/A	0.093^f^ (0.007)
Chief complaint cluster: systemic	N/A	0.009 (0.010)	0.009 (0.010)
NLP cluster: gastrointestinal	0.192^f^ (0.006)	N/A	0.088^f^ (0.007)
Chief complaint cluster: gastrointestinal	N/A	0.305^f^ (0.008)	0.262^f^ (0.008)
NLP cluster: respiratory	0.114^f^ (0.007)	N/A	0.053^f^ (0.007)
Chief complaint cluster: respiratory	N/A	0.121^f^ (0.014)	0.088^f^ (0.014)
NLP cluster: cardiovascular	0.050^f^ (0.006)	N/A	0.030^f^ (0.006)
Chief complaint cluster: cardiovascular	N/A	0.205^f^ (0.009)	0.197^f^ (0.010)
NLP cluster: neurological	−0.015^h^ (0.007)	N/A	−0.002 (0.007)
Chief complaint cluster: neurological	N/A	−0.038^f^ (0.009)	−0.039^f^ (0.009)
NLP cluster: eye, ENT^i^, or skin	−0.134^f^ (0.009)	N/A	−0.061^f^ (0.009)
Chief complaint cluster: eye, ENT, or skin	N/A	−0.302^f^ (0.013)	−0.279^f^ (0.013)
NLP cluster: urological or gynecological	0.055^f^ (0.008)	N/A	0.006 (0.008)
Chief complaint cluster: urological or gynecological	N/A	0.193^f^ (0.012)	0.187^f^ (0.013)
NLP cluster: trauma	−0.129^f^ (0.005)	N/A	−0.098^f^ (0.005)
Chief complaint cluster: trauma	N/A	−0.011 (0.007)	0.013^j^ (0.007)
NLP cluster: psychiatric	0.063^f^ (0.009)	N/A	0.080^f^ (0.010)
Chief complaint cluster: psychiatric	N/A	0.086^f^ (0.012)	0.051^f^ (0.013)

^a^This table presents the results from a linear probability model with the low ESI score indicator as the dependent variable (ESI score of 2 or 3). All models included a set of demographic and administrative covariates.

^b^Observation: 52,222; *R*^2^=0.409.

^c^Observation: 52,222; *R*^2^=0.448.

^d^Observation: 52,222; *R*^2^=0.457.

^e^NLP: natural language processing.

^f^*P*<.01.

^g^N/A: not applicable.

^h^*P*<.05.

^i^ENT: ear, nose, and throat.

^j^*P*<.10.

Of the 12 symptom clusters, 11 (92%) in column 1 had a significant regression coefficient for hospitalization (all but “general organizational”). Eight clusters remained significant even when including the cluster of clinician-determined chief complaints in the model. In the model explaining “inpatient,” in 10 (83%) out of the 12 symptom cluster pairs, the coefficients of the NLP topic clusters showed the same algebraic sign as the chief complaint clusters. In contrast, for 2 symptom cluster pairs, they did not (“general symptoms” and “trauma”). A change in the algebraic sign of either the chief complaint cluster or the NLP topics cluster occurred in 4 cluster pairs when both NLP topics and chief complaints were included in the model (“COVID,” “general symptoms,” “general organizational,” and “respiratory”). We obtained similar results when analyzing the low ESI scores. However, a change in the algebraic sign of a coefficient within solely 1 pair of symptom clusters was noted (“trauma”). Interestingly, the clusters “cardiovascular,” “neurological,” and “trauma” were significantly associated with nonhospitalization, of which “neurological” and “trauma” but not “cardiovascular” were also significantly associated with a lower ESI score.

As a robustness check, we used each of the 3 model specifications to predict the ESI indicator and the inpatient indicator. Using the respective sets of variables of each specification, we used a logistic regression with a 2:1 train-test split to predict both outcomes. Table 7 shows the F_1_-score and area under the curve (AUC) score of these predictions. The results show that the 3 specifications have similar predictive power (an AUC of 0.82-0.84 for “inpatient” and an AUC of 0.90-0.92 for ESI indicator).

The inference and prediction results show that the added value of text in this setting is not by increasing the predictive power of the model, where the outcomes are existing process outcomes (eg, discharge type of severity). Instead, unstructured text allows clinicians to access more granular information to optimize patient flows, which cannot be reflected in the inpatient and ESI indicator outcomes.

In a more granular analysis, we estimated models 1 to 3 with the individual NLP topics and the individual LS groups instead of the clusters previously used. The analysis corroborated our clinical presumptions that, for example, age, admission by an ambulance, and “sepsis” as an NLP topic, as well as “chest pain” for a chief complaint, were associated with low ESI scores (2 or 3) or hospital admission. In contrast, the NLP topic or chief complaint cluster “follow-up” was not. The complete results are provided in Tables S3-S6 in Multimedia Appendix 1.

**Table 7:** Prediction of hospitalization (“Inpatient”) and low Emergency Severity Index (ESI) score of 2 or 3 (“Low ESI score”).

Variable and model		F_1_-score on ones	AUC^a^
**Inpatient**			
	Model 1: NLP^b^ clusters	0.57	0.82
	Model 2: LS^c^ clusters	0.57	0.83
	Model 3: NLP+LS clusters	0.59	0.84
**Low ESI score**			
	Model 1: NLP clusters	0.86	0.92
	Model 2: LS clusters	0.84	0.90
	Model 3: NLP+LS clusters	0.87	0.92

^a^AUC: area under the curve.

^b^NLP: natural language processing.

^c^LS: lead symptom.

## Discussion

### Principal Findings

Our analysis of patient records showed the additional information extracted from unstructured text and its potential usefulness in the clinical context. We demonstrated that the information extracted from NLP features and the physician’s categorization of chief complaints was *complementary*. Indeed, the correlation and consistency between the chief complaint and NLP-derived clusters were low (Table 4). This finding indicates that the free text from the NLP clusters provides additional information than that contained in the symptom clusters from the structured chief complaints.

The complementarity of the information is further emphasized by the results summarized in Tables 5 and 6, and most coefficients remained significant when both types of indicators were included in the model, suggesting that different aspects of patient information appear to be encoded by the 2 approaches. These results support our hypothesis that NLP-derived libraries capture greater depth and breadth of information than a single chief complaint and underscore the relevance of including information captured in unstructured text to address patient populations.

Surprisingly, the “cardiovascular” and “trauma” clusters were not significant features for predicting hospitalization, with “trauma” also significant for predicting a *higher* ESI score. In contrast, the “systemic” cluster, which included sepsis, anaphylaxis, and neoplastic disease, was significant for predicting hospitalization and a lower ESI score, consistent with clinical expectations. Although symptoms suggestive of cardiac dysfunction and trauma may warrant urgent clinical risk assessment, most patients with such complaints would not require hospitalization. Therefore, early allocation of hospital beds for these subgroups is unlikely to reduce overcrowding. Targeting patients with systemic symptoms, in contrast, is likely to do so.

We also proposed a method for analyzing unstructured clinical notes. Our approach has the advantages of speed, simplicity of implementation, and transparency. The speed at which supervised libraries can be assembled is a strength of the proposed approach. A limitation of implementing supervised NLP algorithms in routine decision support is that they are often resource intensive [17]. In our application, it took an untrained clinician only a few days to assemble the entire library.

Furthermore, using NLP as a tool traditionally requires expertise and the ability to master NLP applications. In fields that require years to decades of training, such as health care, professionals cannot be routinely trained to excel in programming. Thus, a further major barrier to the successful implementation of NLP applications in health care is often the usability of NLP applications [18]. Moreover, the flexibility of the method allows easy adaptation of the created dictionaries to analyze new data sets.

Trust is one of the key benefits of clinician involvement in developing proprietary AI models. Indeed, lack of trust is a recognized major limitation that hinders the potential benefits of using AI in routine clinical practice for organizations and patients [19,20]. Owing to the supervised approach, annotated library compilation is comprehensible and transparent; hence, it is trustworthy for clinicians. This may also become an important advantage if regulation on the implementation of AI use in health care tightens in the future.

The limitation of this study is that our approach still requires manual coding. However, future developments in AI may facilitate this step even further. In addition, human bias was possible because the library was compiled manually. In general, an AI-based text analysis does not achieve perfect precision. However, we primarily advocate using free-text analysis for organizational, not clinical, decision support. Therefore, this limitation is not clinically relevant. A further limitation may lie in the fact that the low correlation between the NLP and chief complaint clusters could stem from errors originating from the manual grouping or NLP clustering. However, we believe these results are plausible. Indeed, the chief complaints “fever” and “pain” were included in the cluster “general symptoms,” as were the NLP-extracted tags “fever” and “pain.” However, as only 1 chief complaint could allocated to a patient, during the COVID-19 pandemic, most patients presenting with fever or influenza-like pain would have most likely been categorized as presenting with the chief complaint “COVID.”

### Conclusions

Health care workers on the one side and EHR engineers as well as hospital administration on the other side are caught in a long, ongoing conflict over the extent of structuring the data entered into EHR. Health care workers often argue that entering structured data is a cumbersome task and that the information archived can be of little use in daily clinical practice. In contrast, administrators and EHR engineers often advocate that structuring data is the only reliable solution, enabling a meaningful analysis of the data. Technological advances may help resolve this conflict.

We were able to demonstrate the importance of maintaining free text in EHR. Indeed, using the chief complaints attributed by a physician from a drop-down menu and a corresponding free-text field as a case in point, we were able to show that free text contains a wealth of information that is not routinely captured by structured data.

Moreover, we developed an approach that could enable the information captured in free text to be easily extracted and processed by hospital informatics systems and fed into a workflow, possibly improving the efficiency of patient management.

Therefore, future EHRs should include the possibility of entering free text.

## Appendix

Multimedia Appendix 1 Supplementary file for web-based publication only.

## Abbreviations

AI: artificial intelligence
AUC: area under the curve
ED: emergency department
EHR: electronic health record
ESI: Emergency Severity Index
LS: lead symptoms
NLP: natural language processing
