# Supplementary material for: Additional Value From Free-Text Diagnoses in Electronic Health Records: Hybrid Dictionary and Machine Learning Classification Study
Source: JMIR Med Inform. 2024 Jan 17;12:e49007. doi: 10.2196/49007 (PMC10831590; doi:10.2196/49007)
Supplement: Multimedia Appendix 1 [file medinform_v12i1e49007_app1.pdf]

Supplementary file for: The value added of free text in electronic health records

Tarun Mehra, M.D.[1], Tobias Wekhof, Ph.D.[2] and Dagmar I. Keller, M.D.[3]

December 12, 2023

---

[1]Department of Medical Oncology and Hematology, University Hospital of Zurich (USZ), Zurich, Switzerland, tarun.mehra@usz.ch (corresponding author)

[2]ETH Zurich, Center of Economic Research and University of Zurich, Department of Banking and Finance, Zurich, Switzerland, twekhof@ethz.ch

[3]Department of Emergency Medicine, University Hospital of Zurich (USZ), Zurich, Switzerland, d.keller@klinik-gut.ch

Table S1: Clusters for Chief Symptom

Cluster aggr	Cluster detail	N	% Total	Cluster aggr	Cluster detail	N	% Total
COVID		7623.0	14.60	neurological		5123.0	9.81
	COVID	7623.0	14.60		head ache	1320.0	2.53
general symptoms		7993.0	15.31		neurological	2202.0	4.22
	fever	421.0	0.81		vigilance, desorientation	117.0	0.22
	pain, exxtremities, joints	5590.0	10.70		syncope	419.0	0.80
	pain, other	679.0	1.30		vertigo, dizziness	800.0	1.53
	back pain	650.0	1.24		convulsion	265.0	0.51
	jaundice	37.0	0.07	eye, ENT, skin		1041.0	1.99
	general weakness	528.0	1.01		eye symptoms	426.0	0.82
	anemia	27.0	0.05		hearing/auricular	4.0	0.01
	divers	61.0	0.12		rash	110.0	0.21
general organisational		642.0	1.23		abscess	501.0	0.96
	follow-up	110.0	0.21	urological, gynecological		1206.0	2.31
	referral	9.0	0.02		flank pain	779.0	1.49
	PEP	523.0	1.00		renal impairement	26.0	0.05
systemic		1983.0	3.80		dysuria	262.0	0.50
	infection	1420.0	2.72		urine retention	70.0	0.13
	anaphylaxia, allergy	307.0	0.59		scrotal pain	69.0	0.13
	admission for transplantation	200.0	0.38	trauma		12337.0	23.62
	glycemia	56.0	0.11		wound	3092.0	5.92
gastrointestinal		4063.0	7.78		contact with chemicals	6.0	0.01
	hemoptysis	34.0	0.07		foreign object	48.0	0.09
	melaena	4.0	0.01		bite/stab injury	369.0	0.71
	pain, upper abdomen	1593.0	3.05		trauma, extremities	3670.0	7.03
	pain, lower abdomen	1655.0	3.17		trauma, torso	298.0	0.57
	vomiting/nausea	496.0	0.95		bleeding n.o.s.	659.0	1.26
	diarrhea	228.0	0.44		trauma, head	668.0	1.28
	obstipation	53.0	0.10		burns	364.0	0.70
respiratory		872.0	1.67		fall	3163.0	6.06
	cold/flu-like symptoms	43.0	0.08	psychiatric		1610.0	3.08
	cough	248.0	0.47		intoxication	693.0	1.33
	dyspnea	581.0	1.11		psychiatric	917.0	1.76
cardiovascular		2245.0	4.30				
	chest pain	1574.0	3.01				
	palpitations	192.0	0.37				
	heart rythm	245.0	0.47				
	hypertension	212.0	0.41				
	hypotension	22.0	0.04				

Note: this table presents the distribution of the diagnosis topics obtained with the annotation based on the chief complaint tag. The total number of cases is 52'222 and 11.47% could not be attributed with a diagnosis-topic. ENT: Ear Nose Throat; PEP: Post Exposition Prophylaxis.

Table S2: Clusters for Chief Complaint vs NLP-tagged

LS cluster	N	0 Tags	1 Tag	2-3 Tags	4+ Tag
COVID	7623	9.52	36.23	44.75	9.50
General symptom	7993	24.45	45.73	29.14	0.69
General admin	642	19.63	29.13	51.25	0.00
Systemic clinical	1983	28.39	50.38	20.17	1.06
Gastrointestinal	4063	35.79	38.25	25.15	0.81
Respiratory	872	23.28	51.49	23.97	1.26
Cardiovascular	2245	27.31	54.92	16.88	0.89
Neurological	5123	24.19	44.80	29.30	1.72
Eye ENT Derma	1041	36.50	43.32	19.21	0.96
Gyn Uro	1206	22.89	63.93	12.60	0.58
Trauma	12337	10.93	42.89	44.24	1.94
General psychiatric	1610	16.83	72.92	9.94	0.31
No tag	5994	28.23	46.95	23.22	1.60
One or more LS tags	46228	19.36	44.53	33.48	2.62

Note: this table presents the distribution of the diagnosis topics obtained with the NLP-based text annotation for each chief complaint cluster. The total number of cases is 52'222. ENT: Ear Nose Throat; Gyn: Gynecology; Uro: Urology.

Table S3: Linear Probability Model on Inpatient with NLP Topics

Variable	*Dependent variable: Inpatient*
Administrative	
Age	0.006* (0.0)
Female	-0.035* (0.003)
Nr patients in ED	0.0 (0.0)
Admission: ambulance	0.309* (0.005)
Admission: police	0.118* (0.027)
Coverage: accident, trauma	-0.048* (0.006)
Shift: night	0.012* (0.005)
Shift: late	0.028* (0.004)
Tuesday	0.004 (0.006)
Wednesday	0.004 (0.006)
Thursday	0.011* (0.006)
Friday	0.002 (0.006)
Saturday	-0.012* (0.006)
Sunday	-0.009 (0.006)
Team: SURG	-0.015* (0.005)
Team: PSY	-0.093* (0.015)
Team: NEUR	-0.025* (0.007)
NLP cluster: COVID	
COVID	0.032* (0.019)
NLP cluster: general symptoms	
fever	0.182* (0.008)
Pain	-0.062* (0.006)
general weakness	0.026 (0.042)
back pain	-0.005 (0.018)
NLP cluster: respiratory	
upper airway	-0.173* (0.014)
lower airway	0.154* (0.013)
flu	-0.126* (0.019)
dyspnea	0.12* (0.009)
NLP cluster: systemic	
infection, n.o.s	0.102* (0.011)
sepsis	0.287* (0.034)
anaphylaxia, allergy	-0.145* (0.024)
cancer	0.104* (0.01)
transplantation	0.481* (0.025)
glycemia	0.107* (0.032)
NLP cluster: cardiovascular	
chest pain	0.02* (0.007)
palpitations, arrythmia	-0.038* (0.017)
pulm. embolism	0.035 (0.023)
DVT	-0.094* (0.017)
hypertention	-0.163* (0.019)
NLP cluster: eye, ENT, skin	
epistaxis	-0.136* (0.049)
eye symptoms	-0.112* (0.015)
hearing, auricular	-0.117 (0.088)
skin	0.006 (0.011)
NLP cluster: psychiatric	
intoxication	-0.099* (0.012)
psychiatric	0.058* (0.017)
fear	-0.107* (0.015)
NLP cluster: general organisational	
follow-up, prescription	-0.011 (0.011)
NLP cluster: gastrointestinal	
GI bleed	0.084* (0.021)
abdominal pain	0.119* (0.009)
diarrhea, vomiting, nausea, obstipation	0.026* (0.008)
NLP cluster: neurlogical	
head ache	-0.086* (0.012)
neurological	0.073* (0.01)
vigilance, desorientation	0.109* (0.027)
dementia	-0.045 (0.077)
syncope	-0.099* (0.018)
vertigo, dizziness	-0.11* (0.015)
convulsion	-0.104* (0.026)
NLP cluster: trauma	
wound	-0.04* (0.006)
fracture, luxation	0.055* (0.007)
trauma, head	-0.044* (0.009)
burns	-0.111* (0.032)
fall	0.047* (0.015)
trauma n.o.s	-0.081* (0.005)
bleeding n.o.s	0.027* (0.015)
collision	0.02* (0.011)
traffic	-0.056* (0.022)
NLP cluster: urological, gynacological	
urological, kidney	0.001 (0.008)
pregnancy	0.059 (0.064)
Severity	
non-severe	-0.065* (0.035)
severe	0.019 (0.025)
chronic	0.18* (0.051)
acute	0.139* (0.025)
Intercept	-0.062* (0.009)
Observations	52,222
R^2	0.289

Note: *p<0.1; **p<0.05; ***p<0.01

The dependent variable is an indicator that takes the value of one if the patient had an inpatient stay and zero otherwise. Standard errors are in parenthesis. The data is from Jan 2014 to Sept. 2019. We also included negations for major topics as separate topic variables but did not report them in this table. The model includes year- and weekday fixed-effects. ED: Emergency Department; DVT: Deep Venous, Thrombosis; ENT: Ear Nose Throat; GI: Gastrointestinal.

Table S4: Linear Probability Model on Inpatient with Chief Complaints

Dependent variable: Inpatient

Administrative	
Age	0.007*
	(0.0)
Female	-0.038*
	(0.003)
Nr patients in ED	0.001*
	(0.0)
Admission: ambulance	0.311*
	(0.005)
Admission: police	0.134*
	(0.028)
Coverage: accident/trauma	-0.054*
	(0.006)
Shift: night	0.013*
	(0.005)
Shift: late	0.029*
	(0.004)
Tuesday	0.003
	(0.006)
Wednesday	0.006
	(0.006)
Thursday	0.009
	(0.006)
Friday	0.002
	(0.006)
Saturday	-0.012*
	(0.006)
Sunday	-0.009
	(0.006)
Team: SURG	0.003
	(0.006)
Team: PSY	-0.089*
	(0.017)
Team: NEUR	0.022*
	(0.009)

Chief complaint cluster: COVID	
COVID	0.126*
	(0.007)

Chief complaint cluster: trauma	
wound	-0.075*
	(0.009)
contact with chemicals	-0.222
	(0.154)
foreign object	-0.123*
	(0.055)
bite, stab injury	-0.11*
	(0.021)
trauma, extremities	-0.036*
	(0.009)
trauma, torso	0.035
	(0.023)
bleeding n.o.s.	0.124*
	(0.016)
trauma, head	0.026
	(0.016)
burns	-0.018
	(0.021)
fall	0.038*
	(0.01)

Chief complaint cluster: general organisational	
follow-up	-0.144*
	(0.036)
referral	0.249*
	(0.126)
PEP	-0.083*
	(0.018)

Chief complaint cluster: systemic	
infection	0.083*
	(0.013)
anaphylaxia, allergy	-0.135*
	(0.022)
admission for transplantation	0.583*
	(0.028)
glycemia	0.179*
	(0.051)

Chief complaint cluster: gastrointestinal	
hemoptysis	0.269*
	(0.065)
melaena	0.453*
	(0.188)
pain, upper abdomen	0.056*
	(0.011)
pain, lower abdomen	0.07*
	(0.011)
vomiting, nausea	0.153*
	(0.018)
diarrhea	0.092*
	(0.026)
obstipation	0.028
	(0.052)

Chief complaint cluster: respiratory	
cold, flu-like symptoms	-0.124*
	(0.058)
cough	0.003
	(0.025)
dyspnea	0.209*
	(0.017)

Chief complaint cluster: cardiovascular	
chest pain	-0.024*
	(0.011)
palpitations	-0.115*
	(0.028)
heart rythm	0.057*
	(0.025)
hypertension	-0.199*
	(0.026)
hypotension	0.23*
	(0.08)

Chief complaint cluster: psychiatric	
intoxication	-0.119*
	(0.016)
psychiatric	0.026
	(0.019)

Chief complaint cluster: neurological	
head ache	-0.077*
	(0.013)
neurological	-0.007
	(0.012)
vigilance, desorientation	0.038
	(0.035)
syncope	-0.079*
	(0.019)
vertigo, dizziness	-0.117*
	(0.015)
convulsion	-0.097*
	(0.025)

Chief complaint cluster: eye, ENT, skin	
eye symptoms	-0.145*
	(0.019)
hearing/auricular	-0.206
	(0.188)
rash	-0.154*
	(0.036)
abscess	-0.093*
	(0.021)

Chief complaint cluster: urological, gynacological	
flank pain	-0.044*
	(0.015)
renal impairement	0.391*
	(0.074)
dysuria	-0.028
	(0.024)
urine retention	-0.174*
	(0.046)
scrotal pain	-0.067
	(0.046)

Chief complaint cluster: general symptoms	
fever	0.21*
	(0.019)
pain, exxtremities, joints	-0.052*
	(0.008)
pain, other	-0.059*
	(0.016)
back pain	-0.009
	(0.016)
jaundice	0.495*
	(0.062)
general weakness	0.123*
	(0.017)
anemia	0.325*
	(0.073)
divers	-0.214*
	(0.049)
Intercept	-0.091*
	(0.01)

Observations	52,222
R^2	0.282

Note: *p<0.1; **p<0.05; ***p<0.01

The dependent variable is an indicator that takes the value of one if the patient had an inpatient stay and zero otherwise. Standard errors are in parenthesis. The data is from Jan 2014 to Sept. 2019. We included also negations for major topics as separate topic variables but did not report them in this table. The model includes year- and weekday fixed-effects. ED: Emergency Department; PEP: Post Exposition Prophylaxis; ENT: Ear Nose Throat.

Table S5: Linear Probability Model on ESI score with AI Tags

	Dependent variable: ESI
Administrative	
Age	0.003* (0.0)
Female	0.001 (0.003)
Nr patients in ED	-0.001* (0.0)
Admission: ambulance	0.226* (0.005)
Admission: police	0.151* (0.027)
Coverage: accident/trauma	-0.127* (0.006)
Shift: night	0.066* (0.005)
Shift: late	0.028* (0.004)
Tuesday	0.0 (0.006)
Wednesday	0.007 (0.006)
Thursday	-0.008 (0.006)
Friday	0.01* (0.006)
Saturday	0.0 (0.006)
Sunday	-0.009 (0.006)
Team: SURG	-0.154* (0.005)
Team: PSY	-0.714* (0.014)
Team: NEUR	-0.671* (0.007)
NLP cluster: COVID	
COVID	0.064* (0.019)
NLP cluster: general symptoms	
fever	0.117* (0.008)
Pain	0.007 (0.006)
general weakness	0.012 (0.041)
back pain	-0.004 (0.018)
NLP cluster: respiratory	
upper airway	-0.014 (0.014)
lower airway	0.098* (0.013)
flu	-0.051* (0.018)
dyspnea	0.14* (0.009)
NLP cluster: systemic	
infection, n.o.s	0.021* (0.011)
sepsis	0.108* (0.033)
anaphylaxia, allergy	0.071* (0.024)
cancer	0.09* (0.01)
transplantation	0.019 (0.025)
glycemia	0.111* (0.032)
NLP cluster: cardiovascular	
chest pain	0.059* (0.007)
palpitations, arrythmia	0.127* (0.017)
pulm. embolism	0.141* (0.022)
DVT	-0.221* (0.016)
hypertention	0.119* (0.019)
NLP cluster: eye/ENT/skin	
epistaxis	0.139* (0.049)
eye symptoms	-0.128* (0.014)
hearing/auricular	-0.054 (0.087)
skin	-0.183* (0.011)
NLP cluster: trauma	
wound	0.021* (0.006)
fracture, luxation	0.003 (0.007)
trauma, head	0.052* (0.009)
NLP cluster: general organisational	
follow-up, prescription	-0.063* (0.011)
NLP cluster: gastrointestinal	
GI bleed	-0.12* (0.021)
abdominal pain	0.222* (0.009)
diarrhea, vomiting, nausea, obstipation	0.135* (0.008)
NLP cluster: neurlogical	
head ache	-0.01 (0.011)
neurological	-0.024* (0.01)
vigilance, desorientation	-0.019 (0.027)
dementia	-0.051 (0.076)
syncope	0.151* (0.018)
vertigo, dizziness	-0.078* (0.014)
convulsion	-0.169* (0.026)
NLP cluster: trauma	
wound	0.021* (0.006)
fracture, luxation	0.003 (0.007)
trauma, head	0.052* (0.009)
burns	0.043 (0.031)
fall	0.056* (0.015)
trauma n.o.s	-0.272* (0.005)
bleeding n.o.s	0.075* (0.015)
collision	0.108* (0.011)
traffic	0.088* (0.022)
NLP cluster: eye/ENT/skin	
epistaxis	0.139* (0.049)
eye symptoms	-0.128* (0.014)
Severity	
non-severe	0.014 (0.035)
severe	0.099* (0.024)
chronic	0.067 (0.05)
acute	0.07* (0.024)
Intercept	0.57* (0.009)
Observations	52,222
R^2	0.448

Note: $^{*}p<0.1$; $^{**}p<0.05$; $^{***}p<0.01$

The dependent variable is an indicator that takes the value of one if the patient had an ESI score below 3 and zero otherwise. Standard errors are in parenthesis. ED: Emergency Department; ENT: Ear Nose Throat; DVT: Deep Venous Thrombosis; GI: Gastrointestinal.

Table S6: Linear Probability Model on low ESI score indicator Chief Complaints

	Dependent variable: ESI
Administrative	
Age	0.003*
	(0.0)
Female	-0.006*
	(0.003)
Nr patients in ED	-0.001*
	(0.0)
Admission: ambulance	0.207*
	(0.004)
Admission: police	0.145*
	(0.025)
Coverage: accident/trauma	-0.118*
	(0.006)
Shift: night	0.081*
	(0.005)
Shift: late	0.031*
	(0.004)
Tuesday	-0.001
	(0.006)
Wednesday	0.003
	(0.006)
Thursday	-0.011*
	(0.006)
Friday	0.006
	(0.006)
Saturday	0.003
	(0.006)
Sunday	-0.008
	(0.006)
Team: SURG	-0.094*
	(0.006)
Team: PSY	-0.631*
	(0.016)
Team: NEUR	-0.534*
	(0.008)
Chief complaint cluster: COVID	
COVID	0.235*
	(0.006)
Chief complaint cluster: trauma	
wound	-0.189*
	(0.009)
contact with chemicals	0.127
	(0.142)
foreign object	-0.077
	(0.05)
bite, stab injury	-0.312*
	(0.019)
trauma, extremities	-0.224*
	(0.009)
trauma, torso	0.125*
	(0.021)
bleeding n.o.s.	0.228*
	(0.014)
trauma, head	0.261*
	(0.015)
burns	0.064*
	(0.019)
fall	0.16*
	(0.009)
Chief complaint cluster: general organisational	
follow-up	-0.464*
	(0.033)
referral	-0.15
	(0.116)
PEP	0.456*
	(0.017)
Chief complaint cluster: systemic	
infection	-0.051*
	(0.012)
anaphylaxia, allergy	0.17*
	(0.021)
admission for transplantation	-0.091*
	(0.025)
glycemia	0.215*
	(0.047)
Chief complaint cluster: gastrointestinal	
hemoptysis	0.309*
	(0.06)
melaena	0.185
	(0.174)
pain, upper abdomen	0.245*
	(0.01)
pain, lower abdomen	0.379*
	(0.01)
vomiting, nausea	0.292*
	(0.016)
diarrhea	0.294*
	(0.024)
obstipation	0.194*
	(0.048)
Chief complaint cluster: respiratory	
cold, flu-like symptoms	-0.205*
	(0.053)
cough	0.031
	(0.023)
dyspnea	0.227*
	(0.015)
Chief complaint cluster: cardiovascular	
chest pain	0.239*
	(0.01)
palpitations	0.293*
	(0.026)
heart rythm	0.208*
	(0.023)
hypertension	0.196*
	(0.024)
hypotension	0.129*
	(0.074)
Chief complaint cluster: psychiatric	
intoxication	0.193*
	(0.014)
psychiatric	-0.001
	(0.018)
Chief complaint cluster: neurological	
head ache	0.002
	(0.012)
neurological	-0.115*
	(0.011)
vigilance, desorientation	-0.027
	(0.033)
syncope	0.215*
	(0.018)
vertigo, dizziness	-0.085*
	(0.014)
convulsion	-0.211*
	(0.023)
Chief complaint cluster: eye, ENT, skin	
eye symptoms	-0.361*
	(0.018)
hearing/auricular	-0.602*
	(0.174)
rash	-0.242*
	(0.033)
abscess	-0.223*
	(0.019)
Chief complaint cluster: urological, gynacological	
flank pain	0.317*
	(0.013)
renal impairement	0.265*
	(0.068)
dysuria	-0.205*
	(0.022)
urine retention	0.243*
	(0.042)
scrotal pain	0.132*
	(0.042)
Chief complaint cluster: general symptoms	
fever	0.214*
	(0.018)
pain, extremities, joints	-0.251*
	(0.007)
pain, other	-0.196*
	(0.014)
back pain	0.025*
	(0.014)
jaundice	0.276*
	(0.057)
general weakness	0.129*
	(0.016)
anemia	0.28*
	(0.067)
divers	0.13*
	(0.045)
Intercept	0.487*
	(0.009)
Observations	52,222
R^2	0.513

Note: *p<0.1; **p<0.05; ***p<0.01

The dependent variable is an indicator that takes the value of one if the patient had an ESI score of 2 or 3 and zero otherwise. Standard errors are in parenthesis. ED: Emergency Department; PEP: Postexposure Prophylaxis; ENT: Ear Nose Throat.
